# Ultrasonographic assessment of early leakage in intestinal sutures in dogs

**DOI:** 10.3389/fvets.2023.1094287

**Published:** 2023-03-02

**Authors:** Giulia Costanzo, Nikolina Linta, Edoardo Auriemma, Simone Perfetti, Sara Del Magno, Alessia Diana

**Affiliations:** ^1^AniCura Istituto Veterinario Novara, Novara, Italy; ^2^Department of Veterinary Medical Sciences, University of Bologna, Bologna, Italy

**Keywords:** intestinal anastomosis, ultrasonography, canine, gastrointestinal tract, complication

## Abstract

Intestinal suture dehiscence is one of the most feared complications following gastrointestinal surgery in both human and veterinary medicine, increasing the morbidity and mortality of these patients. Clinical and laboratory early signs of septic peritonitis are not always easily identifiable while prompt treatment should help decrease postoperative morbidity and mortality. The aim of this study is to describe the ultrasonographic (US) features of confirmed leakage of intestinal sutures (LIS) and to evaluate if this imaging technique can be useful as noninvasive tool for the early diagnosis of LIS. Seven dogs developed LIS in a range of three-four days after gastrointestinal surgery and four of these developed a second dehiscence. On B-mode ultrasonography, all intestinal surgical sites were identified and characterized by a bowel focal thickening with reduced or absent wall layering and the presence of hyperechoic, double-walled foci at regular intervals (suture material). Furthermore, hyperechoic linear interfaces associated with dirty acoustic shadowing and comet-tail artifacts crossing the intestinal wall to free-float in peritoneal cavity or in a saccate collection have been documented. On the basis of these preliminary results, canine abdominal ultrasound seems to be a useful diagnostic technique for post-operative monitoring of patients undergoing intestinal surgery, allowing early detection of signs of a LIS, before the patient develops clinical signs of septic peritonitis.

## Introduction

Intestinal suture dehiscence is one of the most feared complications following gastrointestinal (GI) surgery in both human and veterinary patients, increasing the mortality rate in these subjects (1–3). In dogs, dehiscence of intestinal sutures for biopsy, enterotomy or intestinal resection and anastomosis sites often leads to generalized septic peritonitis resulting in a life-threatening emergency (4) with mortality rates ranging between 74 and 85% (5–7). Dehiscence rates in dogs undergoing small intestinal surgery range from 3.4 to 14%. (6, 8–13). Risk factors reported to be associated with GI dehiscence in dogs include preoperative septic peritonitis, preoperative hypoalbuminemia, American Society of Anesthesiologists (ASA) status classification ≥3, intraoperative hypotension, presence of an intestinal foreign body and involved bowel segment (i.e., large intestine) (3, 6, 9, 13, 14). Early clinical and laboratory signs of septic peritonitis are not always easily identifiable while prompt treatment would help decrease postoperative morbidity and mortality (15). In humans, contrast-enhanced abdominal CT scan is the reference diagnostic standard to define anastomotic leakage and its consequences (15). In small animals, abdominal ultrasound (US) and x-rays are the most commonly used imaging techniques to assess changes of the gastrointestinal tract. Ultrasonography is a non-invasive method to evaluate normal and abnormal gastrointestinal tract (16). Ultrasonographic features of short and long term normally healing enterectomy/enterotomy sites were described (17, 18). In particular, a variety of sonographic changes were identified, all of which showed progressive resolution over a 3–9 days period during the normal process of healing. Major changes at the site of enterectomy/enterotomy included pneumoperitoneum, hyperechoic omental/mesenteric fat, abdominal effusion and altered to absent intestinal wall layering. These sonographic features and the timing of examinations can make the differentiation between these surgically induced changes and anastomotic dehiscence challenging (17). To the authors' knowledge, there are no studies describing the ultrasonographic appearance of the gastrointestinal anastomotic dehiscence in dogs. The aim of this study was to describe the US features of confirmed leakage of intestinal sutures (LIS) and to evaluate if this imaging technique can be useful as a non-invasive tool to diagnose LIS earlier.

## Materials and methods

Medical records of dogs referred to the Veterinary Teaching Hospital of Bologna University from March 2016 through September 2021 were reviewed. Dogs with a surgical or necroscopic diagnosis of LIS were selected. Other inclusion criteria were detailed description of clinical signs at the time of admission and of surgical procedure; clinical evaluation and complete abdominal US examination repeated postoperatively every 24 h thereafter until the diagnosis of LIS. At our hospital this ultrasonographic protocol is applied to all patients considered at high risk of dehiscence after intestinal surgery.

For each case the following information were recorded: clinical history, physical examination at the day of hospitalization/surgery, type of surgery (i.e., enterotomy or enterectomy), anastomosis (i.e., latero-lateralis or termino-terminalis) and type of intestinal suture (hand-sutured or stapled), physical examination the day before surgery and at the time of diagnosis of LIS (LIS1) and, if present, at the time of second dehiscence (LIS2), time elapsed between surgery and LIS (days). In particular, clinical signs included mental status (i.e., normal or abnormal), appetite (i.e., absent, reduced, preserved or increased), regurgitation/vomiting, discomfort at abdominal palpation (i.e., present, absent, well-controlled with analgesia), rectal temperature (i.e., hypothermic, normothermic or hyperthermic).

Two-dimensional US examination of the abdomen was performed by sonographers with 20 years of experience using an ultrasound machine (EPIQ5G ultrasound system, Philips Healthcare, Monza, Italy) equipped with micro-convex (5–8 MHz) and linear (5–12 MHz) probes. Ultrasonographic images were reviewed off-line by one of the authors (NL). The following US findings were recorded:

- features of intestinal surgical site (i.e., location of surgical site, intestinal wall thickness and layering, and peristalsis). Measurements of the intestinal wall thickness were acquired perpendicular to the bowel axis from the serosal to the mucosal surface; intestinal wall layering was considered normal if all layers were distinguishable, altered if layers were visible but had changes in thickness or echogenicity, absent if there was complete loss of wall architecture; intestinal motility was subjectively classified as reduced, increased or absent.

- echogenicity of mesenteric fat surrounding the surgical site and throughout the abdomen: subjectively evaluated (e.g., thickened and normal or increased echogenicity);

- pneumoperitoneum and abdominal free fluid effusion: classified as present or absent. If present quantified as mild/moderate/severe.

- focal fluid accumulation adjacent to the surgical site: recorded as present or absent and if present maximum axial diameters were measured;

- gastrointestinal motility: classified as reduced, increased or absent.

- other gastrointestinal changes (e.g., corrugated small bowel loops or fluid filled stomach) and changes to other abdominal organs (e.g., pancreatic or lymph node abnormalities).

## Results

**Figure 1:**
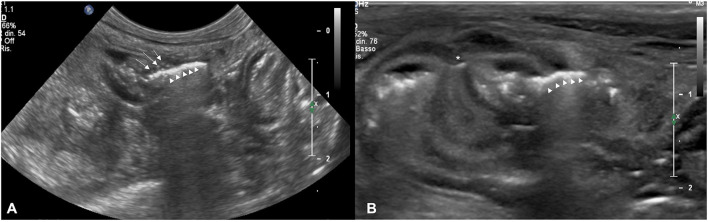
**(A)** Transverse sonogram of an intestinal dehiscence in a dog with termino-terminalis anastomosis obtained with a microconvex probe. Note the mild wall thickening with reduced layering associated with hyperechoic foci (white arrows) coherent with suture material. Hy-perechoic linear interface (withe arrows head) associated with a dirty acoustic shadowing crossing the intestinal wall to free-float in peritoneal cavity is also evident. **(B)** Transverse sono-gram of an intestinal dehiscence in a dog with termino-terminalis anastomosis and serosal patching (white asterisk) obtained with a linear probe. Note the moderate wall thickening with the prominence of muscular layer. Hyperechoic linear interface (withe arrows head) associated with dirty acoustic shadowing and reverberation crossing the intestinal wall at the level of suture site is also evident. The mesentery around the dehiscence is thickened and hyperechoic.

**Figure 2:**
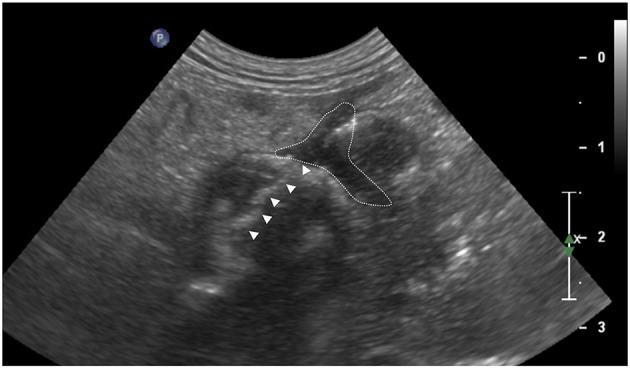
Transverse sonogram of an intestinal dehiscence in a dog with latero-lateralis anastomosis obtained with a microconvex probe. Note the hypoechoic wall thickening with absent wall layering. Irregular hyperechoic linear interface (white arrows head) associated with dirty acoustic shadowing crossing the intestinal wall and reaching a fluid pocket (white dotted line) is also evident. The mesentery around the intestinal loop appears thickened and hyperechoic.

Seven dogs met the inclusion criteria and four of these had a second dehiscence for a total number of LIS 11. The median age was 6 (1-10) years, and the median body weight was 11 (3-35) kg. Information regarding signalment, history, clinical signs, type of surgery and anastomosis are summarized in Table 1. Four dogs (cases 1, 4, 5, and 7) developed a second LIS. Seven out of 11 intestinal dehiscences occurred 3 days after surgery. All the cases examined, both for first and second LIS, showed a very mild deterioration of clinical signs on the day where leakage was suspected by US examination. Results of the physical examination at the day before and at the time of the diagnosis of first and second LIS, the time elapsed between surgery and the LIS (days) are summarized in Table 2. At the US examination all the surgical sites were identified. In all cases a focal thickening of the intestinal wall with reduced or loss of layering ranging from 3.5 to 8 mm with an extension ranging from 4 to 17 mm was observed. Furthermore, in all cases hyperechoic double-walled foci at regular intervals coherent with suture material at the level of the intestinal thickening were detected. In all cases, hyperechoic linear interfaces associated with dirty acoustic shadowing and comet-tail artifacts crossing the intestinal wall and free-flowing in the peritoneal cavity (Figures 1A, B) or reaching a fluid pocket (Figure 2), consistent with generalized or localized free abdominal gas, were present in four and seven cases, respectively. Mild to moderate pneumoperitoneum was observed in all cases. Diffuse bright mesenteric fat was noted in most of the cases of LIS (10 cases). In particular, in 6 cases it was more pronounced at the level of the surgical site and in one case hyperechoic mesenteric fat was noted only adjacent to the surgical site. Other GI sonographic findings included fluid-filled stomach (4 cases), corrugated small bowel loops (6 cases), reduced peristalsis (7 cases) and features of chronic inflammatory intestinal disease (e.g., altered wall layering and diffuse increased mucosal echogenicity) (2 cases). In one dog the remaining tract of the GI tract appeared normal, with normal peristalsis. All dogs show a degree of peritoneal effusion in the post-surgical ultrasonographic follow up. Results of all ultrasonographic findings are summarized in Table 3. In five cases of intestinal dehiscence abdominal fluid was collected revealed a septic neutrophilic inflammation was identified.

**Table 1:** Signalment, clinical signs, type of surgery and anastomosis of a population of dogs with intestinal dehiscence after intestinal surgery.

**Case n°**	**Breed**	**Sex**	**Age (years)**	**Weight (kg)**	**Anamnesis/clinical signs**	**Type surgery**	**Type anastomosis**	**Surgery diagnosis**	**Postoperative course**
1	Yorkshire Terrier	FS	1	3	Obtundation; abdominal pain	Typhlectomy; 2 surgical jejunal biopsies	Termino-terminalis anastomosis hand sutured	Perforation of ceacum	Euthanasia
2	Golden Retriever	FS	10	26	Previous surgery for intussusception; septic peritonitis; obtundation; abdominal pain; hyperthermia;	Jejunal enterectomy	Termino-terminalis anastomosis hand sutured	Intestinal dehiscence of previous surgery for intussusception	Euthanasia
3	Mixed	MN	4	10	Obtundation; anorexia	Jejunal enterectomy	Termino-terminalis anastomosis hand sutured	Occluding intestinal foreign body	Discharge
4	Labrador Retriever	F	5	30	Obtundation; abdominal pain; anorexia; hyperthermia	Jejunal enterectomy	Latero-lateralis anastomosis hand sutured	Occluding intestinal foreign body	Discharge
5	Dachshund	M	6	4	Diagnosis of ibd; obtundation; abdominal pain; anorexia	Jejunal enterectomy	Termino-terminalis anastomosis hand sutured	Jejunal intussusception	Euthanasia
6	Mixed	MN	8	35	Obtundation; abdominal pain; anorexia; mass on abdominal palpation	Jejunal enterectomy	Latero-lateralis anastomosis hand sutured	Occluding jejunal mass	Discharge
7	Mixed	M	6	11	Obtundation; abdominal pain; anorexia; constipation	Colic enterectomy	Termino-terminalis anastomosis hand sutured	Colic perforation for fecaloma	Euthanasia

FS, female spayed; MN, male neutered; F, female intact; M, male intact; IBD, inflammatory bowel disease.

**Table 2:** Clinical data in seven dogs at the day before and at the time of diagnosis of leakage of intestinal sutures (LIS) after intestinal surgery.

**Case n°**	**LIS n°**	**Time surgery LIS (day)**	**Mental Status**		**Appetite**		**Vomiting/Regurgitation**		**Discomfort at Abdominal Palpation**		**Rectal Temperature**	
			**Day before**	**Day of LIS**	**Day before**	**Day of LIS**	**Day before**	**Day of LIS**	**Day before**	**Day of LIS**	**Day before**	**Day of LIS**
1	1	3	AB	AB	Reduced	Reduced	Regurgitation	No	AG	AG	Normothermic	Normothermic
	2	4	AB	AB	Preserved	Preserved	No	No	AG	AG	Normothermic	Normothermic
2	1	3	N	N	Reduced	Absent	No	No	AG	AG	Normothermic	Hyperthermic
3	1	3	N	AB	Preserved	Preserved	No	Regurgitation	AG	AG	Hypothermic	Normothermic
4	1	3	AB	N	Preserved	Preserved	No	No	AG	AG	Normothermic	Normothermic
	2	4	N	N	Preserved	Preserved	No	No	AG	AG	Normothermic	Hyperthermic
5	1	4	N	N	Preserved	Preserved	No	No	AG	AG	Hypothermic	Hypothermic
	2	2	N	AB	Absent	Absent	No	No	AG	AG	Hypothermic	Hypothermic
6	1	3	N	N	Preserved	Preserved	No	No	AG	present	Normothermic	Hypothermic
7	1	3	N	AB	Preserved	Absent	No	No	AG	present	Normothermic	Normothermic
	2	3	AB	AB	Absent	Absent	No	No	present	present	Normothermic	Normothermic

Time surgery LIS, time elapsed between surgery and LIS; AB, abnormal, N, normal; AG; analgesia controlled.

**Table 3:** Ultrasonographic findings observed in each dog at the time (24, 48 h…..) the ultrasound was performed before the time of diagnosis of leakage of intestinal sutures (LIS) after intestinal surgery.

			**Surgical site**									
**Case n**°	**LIS**	**Time US**	**Wall thickness (mm)**	**Extension (mm)**	**Wall layering**	**Peristalsis**	**Changes in the remaining GI tract**	**Bright mesenteric fat**	**Pneumoperitoneum**	**Echogenic peritoneal effusion**	**Fluid-gas filled pocket (mm)**	**Lynph nodes**
1	1	24–48	4	6	Absent wall layering with the presence of hyperechoic, double-walled foci seen at regular intervals (suture material)	Reduced	Fluid-filled stomach	Focal	Mild	Moderate	5 x 3	Normal
	2	24–48–72	3	7	Absent wall layering with the presence of hyperechoic, double-walled foci seen at regular intervals (suture material)	Reduced	Corrugated intestines	Diffused	Mild	Moderate	Absent	Normal
2	1	24–48	5.5	9.5	Absent wall layering with the presence of hyperechoic, double-walled foci seen at regular intervals (suture material)	Reduced	Corrugated intestines	Diffused	Moderate	Moderate	Absent	Normal
3	1	24–48	4.7	5.6	Absent wall layering with the presence of hyperechoic, double-walled foci seen at regular intervals (suture material)	Reduced	Corrugated intestines	Diffused, but more severe focal	Mild	Moderate	10 × 8	Normal
4	1	24–48	6.8	15	Absent wall layering with the presence of hyperechoic, double-walled foci seen at regular intervals (suture material)	Reduced	Normal	Diffused, but more severe focal	Mild	Mild	12 × 7	Jejunal 14 mm
	2	24–48–72	8	17	Absent wall layering with the presence of hyperechoic, double-walled foci seen at regular intervals (suture material)	Reduced	Fluid-filled stomach	Diffused, but more severe focal	Mild	Mild	5 × 3	Jejunal 14 mm
5	1	24–48–72	5	6.4	Absent wall layering with the presence of hyperechoic, double-walled foci seen at regular intervals (suture material)	Reduced	Chronic inflammatory intestinal disease	Diffused	Mild	Mild	11 × 4	Normal
	2	24	4.3	4	Absent wall layering with the presence of hyperechoic, double-walled foci seen at regular intervals (suture material)	Reduced	Chronic inflammatory intestinal disease	Diffused	Mild	Mild	10 × 5	Normal
6	1	24–48	6.6	8	Absent wall layering with the presence of hyperechoic, double-walled foci seen at regular intervals (suture material)	Reduced	Corrugated intestines	Diffused, but more severe focal	Moderate	Mild	Absent	Normal
7	1	24–48	5	10	Absent wall layering with the presence of hyperechoic, double-walled foci seen at regular intervals (suture material)	Reduced	Fluid-filled stomach; corrugated intestines	Diffused, but more severe focal	Moderate	Mild	10 × 2	Normal
	2	24–48	7	10	Absent wall layering with the presence of hyperechoic, double-walled foci seen at regular intervals (suture material)	Reduced	Fluid-filled stomach; corrugated intestines	Diffused, but more severe focal	Moderate	Mild	12 × 5	Normal

## Discussion

In this study we describe the US features of intestinal suture dehiscence. On the basis of our results, abdominal ultrasound is a useful imaging technique for the early diagnosis of LIS. Information acquired allowed to discriminate between intestinal wound dehiscence requiring revision surgery from uneventful healing of intestinal surgical sites.

The population included in this retrospective study consisted exclusively of dogs, whereas no cat undergoing intestinal surgery at our facility subsequently experienced dehiscence of the surgical site during the considered time-period. This finding agrees with others two previous studies reporting an incidence <1% of dehiscence in cats receiving GI surgery (6, 19).

The clinical signs occurring during intestinal dehiscence can be quite variable and non-specific and are usually more severe in those patients with septic peritonitis. The most frequently reported clinical signs in patients with dehiscence include obtundation, abdominal pain, anorexia, vomiting, diarrhea, dehydration, tachycardia and tachypnea. In addition, signs of hemodynamic instability and cardiac arrhythmias are found if septic peritonitis develop (7).

The overall clinical picture at the day of the US suspected dehiscence in all dogs of the present study was almost compatible with a normal post-operative course. It should be noted that because of the use of the post-operative analgesic treatment, clinical signs of post-operative complications such as abdominal pain, tachycardia and tachypnea, could have been masked.

The causes requiring first intestinal surgery in dogs of this study were different and similar to those most frequently reported in the canine literature (e.g., foreign body ingestion, neoplastic mass, intussusception, chronic inflammation mesenteric torsion, trauma and diagnostic biopsy) (20). Furthermore, in all dogs there was at least one of the known preoperative predisposing conditions to the development of dehiscence of the surgical anastomosis (i.e., foreign body ingestion, underlying neoplasm, inflammatory enteritis, pre-existing peritonitis at surgery) that interfere with the healing processes making the suture more fragile (4, 6, 9, 21).

All dogs of the present study developed LIS between the 2nd and 4th day after surgery and in most of them (7/11 cases) LIS arose within 3 days after the surgery, as previously reported (10, 22). This is because the most critical phase of the gastrointestinal wound healing process is the inflammatory one, which occurs in the first 72–96 h after surgery (22).

Regarding US findings of dehiscence, all the dogs of the present study showed some US changes also described in the normal post-operative course such as thickening of the intestinal wall at the level of surgery with altered to absent wall layering, double-wall hyperechoic foci corresponding to the intestinal sutures, hyper-echogenicity of the mesenteric fat (both surrounding the surgical site and throughout the abdomen), fluid-filled stomach, corrugated small bowel loops, reduced peristalsis, abdominal effusion and pneumoperitoneum (17). Mild to moderate pneumoperitoneum was found in all dogs of this study but this finding was not considered pathognomonic for intestinal dehiscence alone as free localized or generalized abdominal gas is described to be a normal postoperative finding decreasing progressively by day 10 (17). Abdominal effusion either localized or generalized was present in all patients between day 2–4 postoperative. Based on previous studies the presence of abdominal echogenic fluid is not significant correlated to peritonitis (17). Abdominocentesis and fluid analysis was recorded in 5/11 cases of dehiscence resulting in septic inflammation and supporting the ultrasonographic diagnosis of LIS. Although visualization of bacteria on cytology of peritoneal fluid could be suggestive of gastrointestinal dehiscence, false positive results can occur as previously described in one study and clinical utility of a positive culture is limited due to delay in results (23). Bacteria in the abdominal cavity postoperatively can originate from the intestinal tract during surgery, from the intestinal tract postoperatively through incisional dehiscence, or from other sources, such as from either an incisional infection or abdominal drain (23).

Hyperechoic mesenteric fat was noted in all dogs both generalized and surrounding the surgical site similar to normal postoperative course in previously described studies, where progressive resolution should be seen by day 10 postoperative (17).

Fluid filled stomach associate with a reduced gastro-intestinal motility could be interpreted as indirect findings of gastro-intestinal perforation but these features are not specific, as suggested in a previous study (24). Corrugated small intestinal segments were also consistent with concurrent enteritis or abdominal effusion, not necessarily related to gastro-intestinal perforation (24). Absent to altered wall layering and focally thickened bowel wall are considered normal changes of enterectomy or enterotomy sites from day 1 postoperative with alterations remaining for months postoperatively (17). All our cases had a focal thickening of the intestinal wall with abnormal layering ranging from 3.5 to 8 mm, similar to ranges previously described in normal healing of enterectomy sites (17). In all dogs hand sutured enterectomies were performed and the intestinal surgical sites were identified ultrasonographically in all cases. In accordance with previously described studies, enterectomies are more consistently visualized than enterotomies due to the circumferential distribution and larger size of the surgical site (17), even though pneumoperitoneum and hyperechoic mesenteric fat could be a limitation in ultrasonographic evaluation of the postoperative abdomen.

In all of our dogs, hyperechoic spots associated with reverberation artifacts at the level of intestinal anastomosis crossing the entire wall from the intestinal lumen to the serosal side were found. On the outer side of the bowel the gas could be confined (or walled-off, reaching a pocket of fluid) or freely spreading in the peritoneal cavity. These findings have never been reported during the normal post-operative course of intestinal surgery (17) and are suggestive of dissecting intramural gas into the intestinal wall. Localized abdominal fluid associated with free gas or small hyperechoic speckles adjacent to perforated gastro-intestinal sites were described to be representative of the leakage of gastro-intestinal content and a pathognomonic sign of perforation (24). This feature was the main discriminating factor in the choice of revision surgery in our dogs even if the clinical condition of the animals were still stable.

Four of the seven dogs included in this study were euthanized on the owner's request because of poor clinical conditions after second dehiscence of the anastomosis. A previous study reported that the development of two post-surgical intestinal dehiscence is associated with a worse prognosis in small animals (25). The remaining three dogs of this study showed a full recovery after discharge and, thus, the mortality rate was 57%. This percentage is lower than the 70–85% range reported in dogs that developed intestinal wound dehiscence after enterectomy for intestinal foreign body occlusion, neoplasia, intussusception, severe enteritis, torsion, and trauma (5, 6, 14). Our finding can be explained considering that most dogs of the present study were referred to revision surgery when their clinical conditions were still stable and no systemic signs of septic peritonitis were present. The overall mortality could only have been lower, and not worsened, if the owners that elected euthanasia would not have done so.

The main limitations of this study are due to its retrospective nature. In some cases, the lack of detailed information regarding laboratory parameters, hypotension during surgery, tension on the edges of the anastomosis or intestinal hypoperfusion state could not exclude that these predisposing factors influenced the development of dehiscence. Finally, the relative low case number of this study may represent a limit, as in order to adequately assess the accuracy of ultrasound as a diagnostic tool for intestinal dehiscence, a larger study population may be required as long as a comparison with dog with a normal post-operative course.

In conclusion, the ultrasonographic diagnosis of LIS may not be simple as several US features found during dehiscence (e.g., pneumoperitoneum, corpuscular abdominal effusion, small fluid collections and peritonitis) are also described in the normal post-operative course. For this reason the experience of the sonographer is a key element. However, small linear interfaces associated with comet-tail artifacts crossing the intestinal wall near the suture and free-flowing in the peritoneal cavity or reaching fluid pocket may be ultrasound features of dehiscence of the intestinal suture.

Our results indicated that US examination performed around 3–4 days after intestinal surgery is recommended for an early detection of LIS even in subjects without overt clinical signs.

## Data availability statement

The original contributions presented in the study are included in the article/supplementary material, further inquiries can be directed to the corresponding author.

## Author contributions

Conceptualization: AD and NL. Investigation: AD, NL, and SP. Data curation and writing—review and editing: GC, AD, NL, SD, EA, and SP. Writing: GC and NL. Supervision: AD and EA. All authors have read and agreed to the published version of the manuscript. All authors contributed to the article and approved the submitted version.
