# Corrigendum: Ultrasonographic assessment of early leakage in intestinal sutures in dogs

**DOI:** 10.3389/fvets.2023.1192801

**Published:** 2023-04-13

**Authors:** Giulia Costanzo, Nikolina Linta, Edoardo Auriemma, Simone Perfetti, Sara Del Magno, Alessia Diana

**Affiliations:** ^1^AniCura Istituto Veterinario Novara, Novara, Italy; ^2^Department of Veterinary Medical Sciences, University of Bologna, Bologna, Italy

**Keywords:** intestinal anastomosis, ultrasonography, canine, gastrointestinal tract, complication

**Error in the Article Type**

In the published article, there was an error in the article type. Instead of Case Report, it should be Brief Research Report.

The authors apologize for this error and state that this does not change the scientific conclusions of the article in any way. The original article has been updated.

